# An exploratory multimodal imaging and clinicopathological strategy for suspected Kümmell’s disease in the absence of MRI

**DOI:** 10.3389/fmed.2026.1871132

**Published:** 2026-07-09

**Authors:** Buqing Ma, Guanxiong Wang, Baokun Lin, Dongxu Yang, Yijing Fang, Yihao Zhu, Zibin Li, Chang Liu, Jie Ding, Jiayang Li, Haoyang Rao, Bin Mai, Zhen Zhang, Guoye Mo, Huizhi Guo, Danqing Guo, Yanhuai Ma, Yuewei Lin, Yongchao Tang, Kai Yuan, Shuncong Zhang, Haishan Li, Yongxian Li

**Affiliations:** 1 The First Clinical College of Guangzhou University of Chinese Medicine, Guangzhou, Guangdong, China; 2 The First Affiliated Hospital of Guangzhou University of Chinese Medicine, Guangzhou, Guangdong, China; 3 State Key Laboratory of Traditional Chinese Medicine Syndrome, The Second Clinical College of Guangzhou University of Chinese Medicine, Guangzhou University of Chinese Medicine, Guangzhou, China; 4 Lingnan Medical Research Center of Guangzhou University of Chinese Medicine, Guangzhou, China

**Keywords:** Kümmell’s disease, magnetic resonance imaging, osteonecrosis, osteoporosis, radionuclide imaging, spinal fractures

## Abstract

**Background:**

Diagnosing Kümmell’s disease (KD) remains a clinical challenge when patients present with contraindications for magnetic resonance imaging (MRI). To address this issue, we propose and make a preliminary attempt at a promising exploratory multimodal diagnostic strategy. It directly combines structural imaging with metabolic data for KD when MRI is contraindicated.

**Materials and methods:**

We evaluated suspected KD patients using a stepwise workflow. Following initial clinical symptom screening, patients underwent plain radiography and high-resolution computed tomography (CT) to detect key morphological signs, such as intervertebral vacuum clefts (IVCs) and fracture-end sclerosis. Finally, we utilized ^99^ᵐTc-MDP bone scintigraphy, namely emission computed tomography (ECT), to assess the actual metabolic status of the affected vertebrae.

**Results:**

CT scans provided conclusive anatomical evidence of vertebral injury and instability. ECT scans revealed metabolic activity, identifying lesions with high metabolic activity (hot areas) or decreased radiotracer uptake intensity (cold areas), corresponding to repair processes and avascular necrosis, respectively. The combination of these multimodal examinations provided convergent imaging evidence supportive of the diagnosis of KD.

**Conclusion:**

For KD patients who are not suitable for MRI, a combination of CT and ECT bone imaging may serve as a feasible exploratory diagnostic approach. This technique may help address some limitations of single-modality assessment by linking anatomical information with metabolic activity, contributing to more accurate diagnosis and more informed clinical decision-making.

## 1 Introduction

Kümmell’s disease (KD) was first described by Hermann Kümmell in 1895 (1). This is a delayed complication of osteoporotic vertebral compression fractures (OVCFs). Clinically, after minor injury, patients typically experience a period of several weeks to several months without symptoms, after which they develop kyphosis (2–4), neurological impairments, or chronic low back discomfort. The existence of intravertebral vacuum cleft (IVC) and the collapse of post-traumatic vertebral avascular necrosis are two important diagnostic indicators (5). Magnetic resonance imaging (MRI) offers significant advantages in soft tissue imaging, providing precise visualization of ligament injuries, disks, and spinal nerves. Moreover, through the “double-line sign” and “fluid sign,” it can reliably distinguish hematoma and edema in the fractured vertebrae, which is of great diagnostic significance for KD stage and the type of IVC contents (6, 7). However, accurate diagnosis of KD remains difficult in clinical practice due to the insidiousness of the disease and the presence of physiological contraindications to MRI in some individuals. This study proposes and validates a multimodal diagnosis method combining X-ray, CT and emission computed tomography (ECT) bone imaging, aiming to solve the clinical problem of the lack of standardized diagnostic pathways in patients with suspected KD and contraindications to MRI examination. This method aims to address deficiencies in the current diagnostic process by integrating comprehensive morphological evidence from X-ray and CT with bone metabolic function data from ECT, aiming to close gaps in current recommendations and provide a structured exploratory diagnostic workflow for patients who are not suitable for MRI.

## 2 Materials and methods

This study was conducted in accordance with the Declaration of Helsinki and approved by the Institutional Ethics Committee of The First Affiliated Hospital of Guangzhou University of Chinese Medicine (ID: K-2026-010). Detailed ethical declarations and patient consent details are provided in the Ethics Statement section at the end of this article.

### 2.1 Study design and case selection

This study was a single-center, retrospective exploratory study. Researchers retrospectively screened approximately 20 patients at our center clinically suspected of having KD and whose MRI examinations were limited or contraindicated. Since this study aims to demonstrate the multimodal diagnostic process rather than validate diagnostic efficacy, two representative cases with complete clinical data, complete X-ray/CT/ECT imaging, available pathological evidence, and follow-up information were selected according to preset criteria for image-pathology correlation analysis. These two cases represent the active stage of KD repair and the stage of advanced ischemic necrosis/pseudoarthrosis, respectively (Table 1).

**Table 1:** Baseline characteristics of the retrospectively screened suspected KD cohort.

Characteristics
Number of patients
Age, mean ± SD or median (range)
Female, *n* (%)
Osteoporosis severity/BMD if available
Main symptoms
Vertebral levels involved
MRI contraindication/non-feasibility reasons
Patients with CT evidence of IVC
Patients undergoing ECT
Patients with pathology available

KD, Kümmell’s disease; SD, standard deviation; BMD, bone mineral density; IVC, intravertebral vacuum cleft; ECT, emission computed tomography; CT, computed tomography.

### 2.2 Initial screening based on patient history and physical examination

We concentrated on older women with severe osteoporosis (8) and evaluated their history of minor trauma, asymptomatic intervals, and recurring thoracolumbar pain. Upon the exclusion of prevalent illnesses such as uncomplicated trauma, disk herniation, ankylosing spondylitis, metastatic tumors, and traumatic myositis, KD should be seriously considered. Imaging investigations were essential at this juncture.

### 2.3 Morphological imaging evaluation

Standard radiographs were used for initial screening, which showed dynamic vertebral changes in flexion and hyperextension positions. The specific morphology of vertebral fracture lines, the three-column anatomical structure, facet joint fractures, and the degree of spinal canal stenosis were then evaluated using high-resolution CT imaging with three-dimensional reconstruction. Notable results included the presence of fracture end sclerosis and IVC.

### 2.4 Bone metabolic function assessment using radionuclide imaging

Whole-body bone scintigraphy employing ECT was recommended for patients with worrisome morphological imaging results who were unable to have an MRI. As a functional radionuclide imaging technique, it was useful for identifying KD in individuals who are unable to undergo MRI since it not only showed skeletal morphology but also early blood flow and metabolic condition (9). The absorption of the tracer in specific bone regions (^99^ᵐTc-MDP) is directly proportional to local blood flow and bone mineral metabolism, achieved by ion exchange and chemical adsorption processes, leveraging the tracer’s capacity to disperse with blood flow and accumulate in bone tissue (10, 11).

### 2.5 Pathological validation

According to findings by Cardis and Ranjan et al. (12, 13), macroscopically, damaged vertebrae seemed to be wedge-shaped, and microscopic examination revealed pale ischemic bone fragments and thin, irregular osteoporotic plates. In the initial phases of the damage, deleterious lesions were observable in the affected vertebrae. A recent hemorrhage was seen within the closed medullary cavity, surrounded by aggregates of foamy histiocytes. In the final stage, there was an absence of osteoblast or osteoclast activity in the spinal marrow, characterized only by ossification and fibrinous necrosis. This was indicative of the final stage clinical aspect of KD, which was recurring non-union fractures. The diagnosis of KD was corroborated by the histological features observed in the case patient’s biopsy images, aligning with the research findings. Pathological evidence was not routinely obtained for all suspected KD patients in this study. Tissue specimens are selectively obtained only when patients need surgery for clinical treatment or when further differentiation of tumors, infections, or other lesions is needed. Pathological results were used to supplement and validate the pathophysiological basis of imaging inferences, rather than as a unified screening criterion for this study. Pathological assessment is completed by our hospital’s pathologists according to routine clinical procedures; this study did not implement study-level imaging-pathology double-blind interpretation. Given the invasive nature of pathological material collection, its value in KD is better regarded as important supportive evidence available in specific clinical scenarios, rather than a standard reference standard applicable to all patients.

In this study, two selected representative cases underwent surgical intervention, and vertebral body tissue samples were successfully obtained intraoperatively. The histological features observed in the biopsy images of both patients confirmed osteonecrosis and specific repair responses under the microscope, consistently confirming the diagnosis of KD and consistent with our multimodal imaging results.

### 2.6 Descriptive exploratory analysis

As this study was an exploratory case presentation, only 2 representative cases were included, and no inferential statistical analysis was performed. This study mainly uses descriptive methods to comprehensively analyze clinical manifestations, X-ray/CT/ECT imaging features, and pathological results, with a focus on elaborating the image-pathology correspondence between different modalities.

## 3 Results

**Figure 1:**
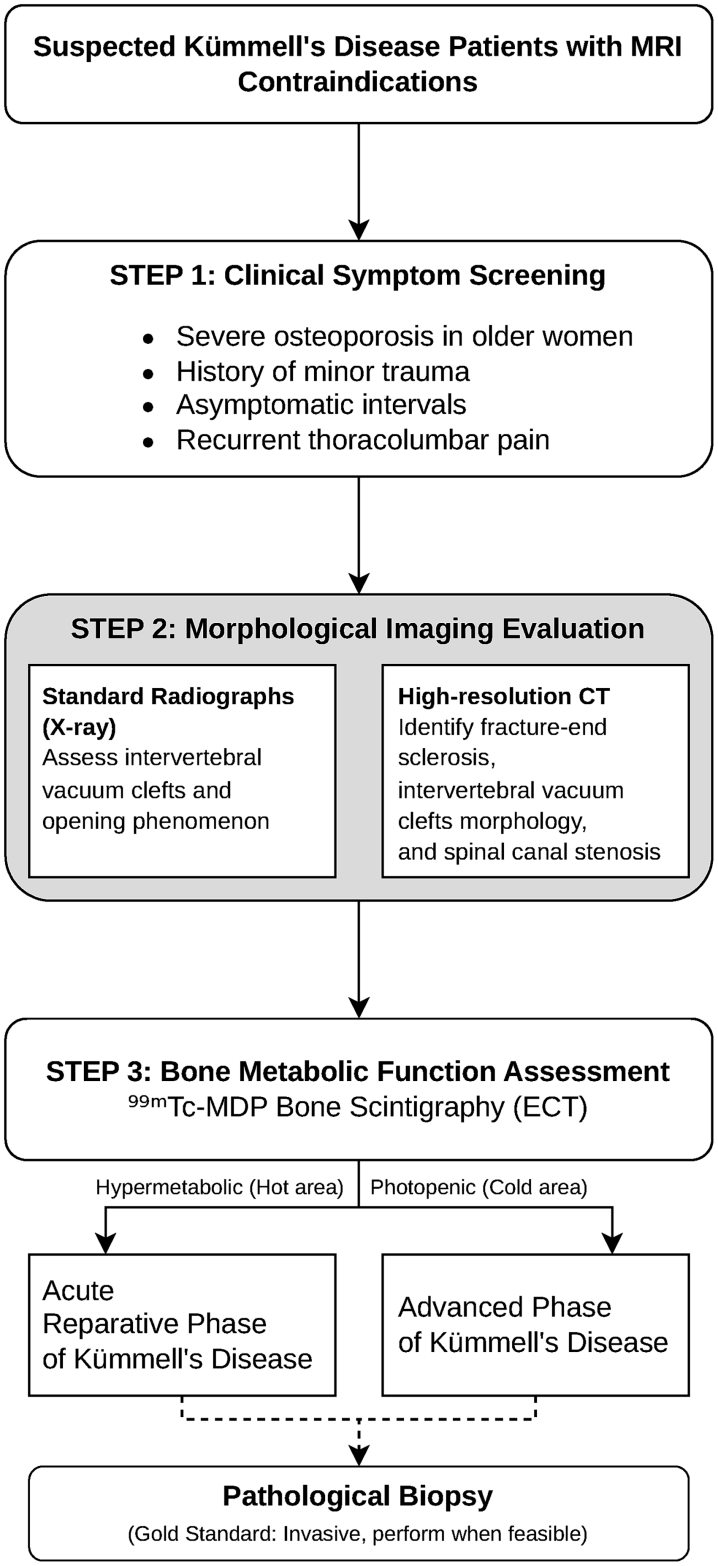
Diagnostic process for suspected KD patients with contraindications to MRI.

By applying the multimodal protocol (summarized in Figure 1), we detail the characteristic diagnostic findings through our representative cases. Based on the diagnostic process described above, the individual examination steps present the following characteristic results in the diagnosis of KD.

### 3.1 X-rays and CT imaging features

**Figure 2:**
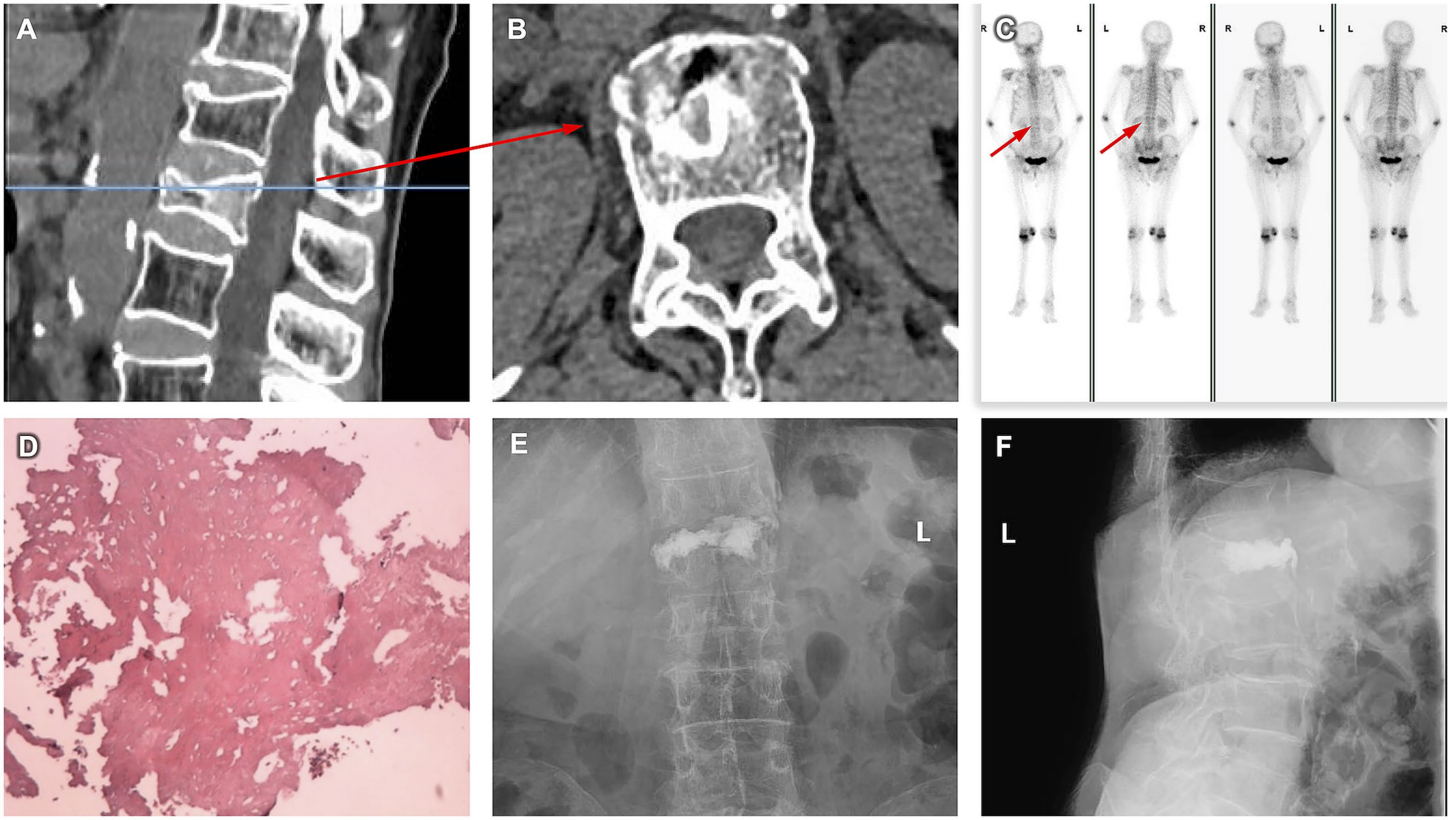
Multimodal imaging and clinical-pathological features of KD in the reparative phase (Case 1). **(A,B)** Preoperative plain radiographs. The anteroposterior and lateral views reveal generalized osteopenia and a significant anterior wedge compression deformity of the L1 vertebra, accompanied by focal kyphotic angulation. **(C,D)** Preoperative sagittal and axial CT scans. A distinct, horizontally oriented intravertebral vacuum cleft (IVC) is clearly visible within the collapsed L1 vertebral body. This radiolucent shadow represents the accumulation of nitrogen gas within the necrotic cavity. The surrounding cancellous bone exhibits reactive osteosclerosis and trabecular impaction, without significant retropulsion of the posterior vertebral wall into the spinal canal. **(E)** ^99^ᵐTc-MDP whole-body bone scintigraphy. Note the intense, diffuse radiotracer accumulation (hypermetabolic “hot area”) spanning from L1 to L4. Pathologically, this reflects intense local bone turnover and osteoblast aggregation attempting to reconstruct the interface between living and necrotic bone during the acute/subacute reparative phase. **(F)** L1 vertebral biopsy histology. The image demonstrates typical trabecular collapse and severe avascular necrosis. The visual field is dominated by homogeneous, eosinophilic necrotic bone tissue. Notably, the bone lacunae are entirely empty without nuclear staining, which is a hallmark of acellular necrosis. There is a complete absence of osteoblast or osteoclast metabolic activity. Furthermore, the normal hematopoietic and fatty marrow architecture has been entirely obliterated and replaced by amorphous necrotic debris and fibrotic exudate. These findings provide direct histological evidence of complete local blood supply interruption. No tumor cells or tuberculous granulomas are present. Postoperative anteroposterior and lateral X-rays demonstrate restored vertebral height and well-distributed bone cement.

**Figure 3:**
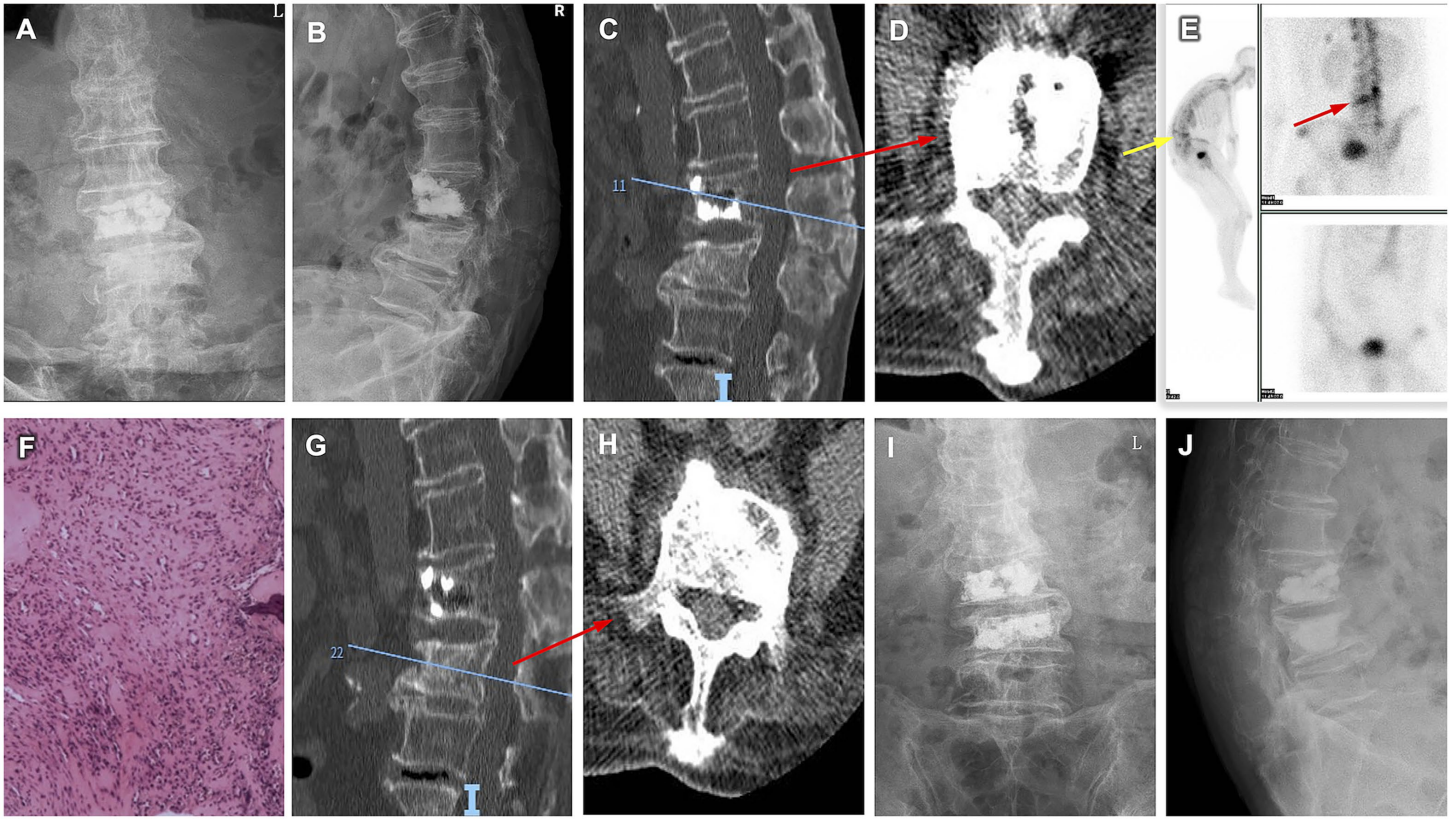
Multimodal imaging and clinical-pathological correlation of KD in the advanced avascular necrosis phase (Case 2). **(A,B)** Follow-up plain radiographs. Anteroposterior and lateral radiographs taken 19 months after previous cement augmentation surgeries reveal severe structural deterioration at L3, characterized by marked anterior column collapse and progressive local kyphosis. **(C,D)** High-resolution CT scans at the 19-month follow-up. The sagittal and axial reconstructions demonstrate a prominent IVC sign (red arrow) at L3, surrounded by extensive, dense osteosclerosis (eburnation) of the fractured endplates. The blue lines highlight the severe local kyphotic Cobb angle. The intravertebral gas and severe wedge deformity indicate profound biomechanical instability and pseudoarthrosis formation. **(E)** Bone scintigraphy. The scan shows strong focal tracer uptake at the previous surgical sites. Crucially, L3 displays a characteristic “hot ring” of peripheral osteoblastic activity surrounding a central photopenic area (“cold center,” indicated by yellow arrow). In terms of pathological significance, the “cold center” corresponds to the avascular necrotic cavity where tracer uptake remains weak due to absent bone repair. Conversely, the surrounding “hot ring,” the photon-enhanced ring region outside the cold center (“hot ring,” red arrow), indicates intensely active local bone turnover induced by constant friction and microfractures at the pseudoarthrosis interface. **(F)** Histology of the L3 lesion. The specimen displays an active but disorganized tissue remodeling response at the osteochondral margin, typical of a fibrous pseudoarthrosis interface. The field is dominated by dense, haphazardly arranged fibrous connective tissue characterized by abundant fibroblast proliferation and an interwoven collagen network. Interspersed microvessels and granulation tissue indicate an active but “frustrated” attempt at revascularization. This exuberant fibrous proliferation around necrotic bone fragments is a classic microscopic manifestation of halted bone healing secondary to chronic mechanical micro-motion and physical friction, resulting in a fragile fibrous union rather than robust bony fusion. **(G,H)** CT images of L4. The sagittal and axial views confirm progressive adjacent segment disease with a new IVC (red arrow) and cortical disruption, exacerbating the overall structural collapse. **(I,J)** Postoperative plain radiographs. Anteroposterior and lateral X-rays after the subsequent L4 salvage surgery display the stable distribution of bone cement filling the clefts and partial correction of the kyphotic deformity.

The IVC (14) often appears in the thoracolumbar segment (T12-L1) (15) and presents as linear or crescent-shaped translucent shadows within the collapsed vertebral body, which is the primary sign of KD on plain radiographs (see Figures 2E, F and 3A, B, I, J). The “opening phenomenon” was commonly observed in X-rays taken in hyperextension and overflexion positions (16). This phenomenon provided direct evidence of vertebral instability by reflecting the mobility of the vertebral pseudoarthrosis in conjunction with the dynamic alterations of the IVC. This demonstrated the unique diagnostic value of X-rays in KD (16, 17). Basic X-rays may provide a first assessment of the injured vertebrae and joint mobility; nevertheless, they were insufficient for diagnosing KD.

CT scanning employed three-dimensional reconstruction technology, which clearly illustrated the precise morphology of spinal fracture lines and the three-column anatomical structure, significantly helped in the assessment of fracture severity, extent of involvement, and subtle lesions (18). They could accurately assess the extent of spinal canal stenosis and spinal cord compression, as well as clearly illustrate facet joint fractures, the displacement and distribution of bone fragments, and the stability of fractured vertebrae (19). CT reconstructions can clearly illustrate IVC and sclerosis at the fracture ends (see Figures 2A, B and 3C, D, G, H), in addition to revealing substantial osteoporosis in the vertebrae surrounding the sclerotic ends, along with concomitant segmental disk degeneration. Furthermore, certain patients presented with disk calcification, disk effusion, or disk herniation during initial evaluation (20, 21). These imaging features were closely related to the pathological processes associated with vertebral ischemia and necrosis, poor repair, structural collapse, and osteoporosis in patients with KD, and were a solid basis for diagnosing KD.

### 3.2 ECT bone imaging features of osteonecrosis

ECT bone imaging overcomes the limitations of CT and X-rays in detecting metabolic activity by demonstrating the “active” affected vertebrae (9). After the bone tracer ^99^ᵐTc-MDP was injected intravenously, bone metabolism was assessed locally using imaging results that demonstrated tracer uptake: radioactive “hot areas” (22) indicated regions with active bone formation (such as fracture healing or osteogenic tumors); on the other hand, radioactive “cold areas” (23) indicated regions with impaired blood supply or osteolytic lesions. This imaging technique showed exceptional sensitivity in detecting early skeletal lesions and hidden fractures due to its reliance on skeletal physiological metabolism (24). The diagnosis of KD indicated enhanced tracer uptake, creating a hypermetabolic “hot area,” which showed that the patient’s bone tissue was either in the acute phase of the illness or in the fracture healing phase, signifying a recent bone lesion or a recent vertebral fracture (25, 26). As shown in case 1, high radiotracer uptake at L1 levels reflects strong active bone metabolism during the repair phase (Figure 2C). Conversely, diminished uptake resulting in hypometabolic “cold areas” strongly indicated spinal ischemic necrosis with halted recovery of bone, evidenced by nonunion of prior fractures (27). Conversely, as shown in case 2, the L3 vertebral body shows a characteristic central photon reduction zone, or “cold center,” surrounded by a peripheral margin of increased uptake, indicating the presence of advanced avascular necrosis and pseudoarthrosis (Figure 3E). This was highly consistent with the pathological process of vertebral necrosis and pseudoarticular formation in late-stage KD, and the diagnosis of bone tumor lesions, or advanced vertebral ischemic necrosis in KD was considered.

Previous studies had confirmed that in patients with IVC (28–30), bone scans could show abnormal uptake of collapsed vertebrae, whether it was hot areas formed by increased uptake in the early repair stage or cold areas caused by late-stage osteonecrosis, which provided a functional basis for diagnosis.

### 3.3 Pathological features of spinal fractures

Histopathological examination showed that the injured vertebral body was macroscopically wedge-shaped. Under the microscope, early lesions showed vertebral destruction, fresh bleeding in the closed bone marrow cavity, and foamy tissue cell clusters. In the late-stage, it manifested as ossification and fibrinous necrosis in the bone marrow, and a lack of osteoblast or osteoclast activity (24). This characteristic of “weak bone repair response and significantly lower regenerative ability than normal fracture healing” verified the clinical mechanism of nonunion of repeated fractures in KD (31). Specifically, the biopsy results of the two representative cases were highly consistent with their respective radiological phenotypes. The active fibrosis in Case 1 corresponded to the hypermetabolic ECT findings (Figure 2D), while the avascular necrosis cavity and pseudoarthrosis in Case 2 directly corresponded to the ECT cold center and CT vacuum gap (Figure 3F). This consistent clinicopathological correlation has clinical value in diagnosing KD.

## 4 Discussion

### 4.1 Limitations of single-modality methods

In an aging society, the incidence of delayed, refractory osteoporotic vertebral compression fractures (OVCFs) is continuously rising. Its hidden course and high misdiagnosis rate make diagnosis extremely challenging (32, 33). Traditional diagnostic methods for KD mostly rely on a single primary imaging method, each with its limitations.

MRI is currently recognized as an important standard for diagnosing and staging KD. Its outstanding soft tissue contrast enables early myeloedema detection through short-time reversal recovery (STIR) sequences and clearly maps the boundaries of bone necrosis using characteristic T2-weighted “fluid signs” and “double-line sign” (34, 35). However, MRI also has certain limitations in diagnosing KD; For example, characteristic edema signals may dissipate during the chronic, delayed KD stage (more than 3–4 months), potentially leading to false negative results (36). Additionally, MRI findings may be ambiguous in distinguishing between symptomatic microfractures and asymptomatic severe degenerative changes common in the elderly (37).

Due to certain contraindications, such as implantation of metal devices or inability to tolerate prolonged scans, clinical application of MRI is often limited (38). When MRI scans are not possible, traditional single-modal alternatives such as X-rays and CT scans have significant diagnostic blind spots and cannot clearly and comprehensively diagnose diseases. Although conventional imaging methods such as X-rays and high-resolution CT scans can clearly show vertebral wedge deformities, they usually cannot distinguish between “plastic deformities” caused by healed, metabolically inactive fractures and mobile pseudoarthrogenesis (39). Additionally, the contents inside the IVC (gas or liquid) are dynamically replaced by the negative pressure generated by the patient’s position and the force exerted by the patient (40). Due to the instability of this characteristic morphology, relying solely on CT to observe gas density can easily lead to false negatives (41). Therefore, relying on morphological changes of the vertebral body to determine the “responsible vertebra” leads to a high rate of clinical misdiagnosis and exposes patients to unnecessary surgical interventions (42).

In contrast, ECT independent functional imaging relies on high sensitivity to osteoblast activity and osteoclast-mediated remodeling changes, but lacks anatomical and morphological specificity. During the natural remodeling process of standard osteoporotic fractures, increased radiotracer uptake can last for 6 to 24 months, making it difficult for ECT to distinguish newly emerging KD lesions from slowly healing older fractures, and its accuracy in diagnosing patients with multiple osteoporotic vertebral fractures is much lower than with MRI (43, 44).

Therefore, when MRI cannot be used, relying solely on morphological or physiological metabolic functions inevitably leads to mistreatment or misdiagnosis of metabolically inactive vertebral deformities or misjudgment of the “responsible vertebra,” affecting diagnosis and treatment outcomes (45).

### 4.2 Diagnostic value and complementary synergy of multimodal methods

To overcome these limitations, this study explores a multimodal X-ray, CT, and ECT strategy as partial alternatives for patients with MRI contraindications. This method focuses on exploring morphological and metabolic imaging in spinal disease lesions, aiming to combine the advantages of several imaging techniques using multimodal imaging. In this mode, high-resolution CT plays an indispensable role. High-resolution CT, with its clear skeletal anatomical details, can capture IVCs and detect whether the pyramid is accompanied by severe edge sclerosis and pedicle ossification, which is a highly specific marker of KD and is important for differentiating acute osteoporotic fractures from naturally healing old fractures (17, 18). More importantly, the “vacuum” phenomenon observed by CT and the “fluid sign” detected by MRI to some extent represent the same underlying pathological features (microinstability at the KD diseased joint interface). Its manifestation changes with the patient’s position. Linn and colleagues (46) found that the contents of the internal vertebral fissures change dynamically: when stretched in the supine position, the body is mainly gas-based, but over time, fluid accumulates within the same fissure (40). This position-dependent variation means CT scans can be performed immediately after the patient is supine, detecting vacuum signs that MRI may miss when prolonged supine positions cause fluid accumulation and signal blurring. In contrast, due to this signal loss, MRI has only 50% sensitivity for detecting disk vacuum signs. This dynamic variability further indicates that when MRI signals are abnormal or unavailable, direct visualization of vacuum cracks by CT becomes an extremely feasible structural marker.

After CT provides structural specificity, ECT offers key metabolic validation. A typical feature of KD is delayed onset (asymptomatic periods lasting from weeks to months). When assessing late, late-stage lesions with longer disease courses and pseudoarthrogenesis (highly active local bone turnover), ECT provides excellent evidence of metabolic activity. Unlike MRI fluid signals, which are highly susceptible to changes in position, ECT can directly and stably form chemisorption images of bone cells for ^99^ᵐTc-MDP, identifying bone resorption at the functional metabolic level. Necrotic areas (cold areas) or active bone-forming regions (hot areas) provide important information about the metabolism of the affected vertebrae. Among patients confirmed by CT for IVC, studies have found that the presence of hypermetabolism on ECT is highly pathologically consistent with the edema or fluid signals detected by MRI (36, 43). Therefore, the combination of CT morphological features and ECT bone metabolism features can be regarded to some extent as a diagnostic basis like the “double-line sign “of MRI, identifying abnormal morphological structures of the diseased vertebral body and pathological evolution of internal vascular necrosis and surrounding granulation tissue proliferation, thereby providing alternative diagnostic criteria (47).

### 4.3 Insights into pathophysiology based on ECT performance results

The unique local radioactive abnormal aggregation pattern shown by ECT is not merely a macroscopic structural anomaly; it also truly reflects the dynamic and phased disruption of the internal bone immune microenvironment within vertebrae, linking macroscopic biomechanics with pathophysiological states. During the acute or subacute repair phase (as shown in Case 1), bone scans usually show diffuse hypermetabolic “hot areas” (48, 49). After initial mechanical damage and transient ischemia of trabecular structures, the vertebral body initiates a strong compensatory repair response. Osteoblasts and osteoclasts gather at the junction of living and necrotic bone. This extensive and active bone turnover aims to reconstruct collapsed trabecular bones and massively accumulate ^99^ᵐTc-MDP tracer, thereby creating a large hot area in bone scintillography. When the lesion progresses to the advanced stage of avascular necrosis (as shown in Case 2), this repair process is hindered and confined to specific areas. Complete ischemia eventually occurs in the central area of the collapsed vertebra (50).

Uptake results from cold regions mainly appear in late KD stages. Anatomically, mechanical injury to fragile sites (such as the foramen of the vertebral body) can damage the local microvascular network, triggering a vicious cycle of “ischemia to fracture to ischemia” (51). This structural disruption and disintegration have a significant impact on bone differentiation, leading to complete immune suppression and halted bone repair. At this time, mesenchymal stem cells in the microenvironment are severely depleted, completely blocking normal bone repair pathways (52). Vertebral body repair is poor in the late stage of ischemic necrosis, with the central region occupied by necrotic cavities, and tracer uptake in this area is relatively weak, forming the unique “cold center” of ECT. Meanwhile, the lesion progresses to fibrous pseudoarthrosis. With spinal movement, the periodic opening and closing of the fissure (like a bellows suction effect) produces chronic, low-intensity mechanical micromovements and continuous friction at the interface, which induces a strong inflammatory response, thereby promoting fibrocartilage proliferation and plasma exudation (53). Instead of forming a strong, normal bony fusion, it forms a structurally fragile fibrous union. This persistent, abnormal local bone turnover, characterized by delayed callus mineralization and excessive braided bone formation, strongly binds to the ^99^ᵐTc-MDP tracer, creating a highly metabolized “hot ring” on ECT (31). Therefore, anatomical evidence of delayed healing of unstable diseased vertebral bodies (CT IVC features) and metabolic evidence of impaired repair (ECT metabolism) jointly establish a connection between morphology and physiology.

Moreover, capturing this abnormal metabolic stress distribution through ECT has prognostic value. ECT can visualize the biomechanical stress distribution around the collapsed vertebrae, and even without obvious CT morphological fractures, increased uptake of adjacent segments may serve as an early warning signal for impending fractures (54). This information can guide surgical teams in preoperative planning to consider preventive reinforcement or immobilization of metabolically active but morphologically intact adjacent segments, thereby extending the diagnostic process into treatment decisions.

### 4.4 Differential diagnosis based on clinical indicators and histological features

During diagnosis, in addition to routine examination indicators, other differential diagnostic indicators should also be considered. Zhu (55) found that compared to OVCF patients, KD patients often exhibit decreased albumin levels, elevated serum phosphorus and parathyroid hormone levels, and are more prone to hypercalcemia. While abnormal ECT uptake is not specific to KD, tumors and infections can also present as “hot areas.” However, when combined with CT findings of IVC involvement and sclerotic margins, tumors or tuberculosis can be effectively ruled out. If clinically necessary, histopathological examination can be performed to assess disease progression: the general manifestations in the acute phase of vertebral body fractures include multiple hemorrhagic foci within the cancellous bone and vertebral displacement, with aggregation of osteoblasts and osteoclasts, accompanied by bone frame atrophy (56); The hallmark features of the late KD stage are osteoclast-mediated bone resorption and formation of spinal pseudoarthrosis. Compared to normal fracture healing, regenerative capacity is significantly reduced at this stage, and bone repair responses are extremely weak (57).

### 4.5 Limitations and outlook

Although the proposed multimodal strategy showed significant pathological correlation, this study was essentially an exploratory descriptive analysis with a limited sample size. Furthermore, this study did not directly compare with MRI, so it cannot assess the performance of the proposed multimodal method relative to MRI in terms of diagnostic confidence or accuracy. Given that MRI remains the most important soft tissue and bone marrow signal imaging tool for KD assessment, the results of this study should not be interpreted as a substitute for MRI, but rather as an exploratory supplementary approach in cases where MRI is contraindicated or not feasible. In this method, pathological biopsy serves as the final, though not mandatory, supplementary validation tool. Pathological biopsy is an important supportive method for diagnosing KD, but due to its invasive nature, it cannot be used as a routine screening method. When surgical intervention is clinically needed, intraoperative histological sampling can be performed to verify consistency of diagnostic results. Additionally, this study focused on elderly female patients (due to their higher symptom positivity rates), but KD also occurred in other populations, which may introduce selective bias and limit the general applicability of the findings.

Additionally, our functional assessments were primarily based on planar bone scintigraphy. Routine SPECT/CT was not available for this historical cohort. Future research must compare this technique with standard MRI. These trials require larger sample sizes and prospective designs. Researchers must also develop clear guidelines for functional scan analysis. Specifically, doctors need standard rules to distinguish between metabolically active “hot zones” and ischemic “cold areas”. Moreover, specific quantitative indicators can make this approach more objective. As advanced hybrid imaging becomes more accessible, we strongly advocate for the use of SPECT/CT over planar scintigraphy. Not only does SPECT/CT provide superior anatomical-functional correlation for precise lesion localization, but clinicians can also measure standardized uptake values during SPECT/CT to quantitatively differentiate metabolically active healing from avascular necrosis. For example, clinicians can use quantitative SPECT/CT technology to extract standardized uptake (SUV) of lesions, combined with Hounsfield units (HU) from CT to construct dual quantitative matrices of morphology and metabolic activity, thereby objectively quantifying KD pseudo joint instability. Hospitals need solid evidence before applying this method in daily practice. Ultimately, while preliminary clinical observations indicate that the consistency between CT-detected IVC signs and ECT metabolic activity offers a promising diagnostic alternative, solid evidence is still required before daily clinical application. Further large-scale prospective studies are imperative to refine this strategy into a universally standardized diagnostic framework, demonstrating its reliability in guiding treatment decisions and predicting long-term patient outcomes.

## 5 Conclusion

When MRI has physiological contraindications or cannot be performed, accurately diagnosing KD and identifying responsible lesions is a real clinical challenge faced by spinal surgery. To address this clinical gap, this study proposes an exploratory multimodal diagnostic strategy that combines X-ray, high-resolution CT, and ECT radionuclide bone imaging. By analyzing cases at different pathological stages, this study preliminarily demonstrated the complementarity between “X-ray and CT anatomical information” and “ECT metabolic function information”: X-ray and CT can identify morphological markers of pseudoarthrosis such as intravertebral vacuum clefts (IVC); while ECT reflects the blood supply status and repair response of the local bone microenvironment. By combining histopathological research and characteristic uptake images on ECT, physiological mechanisms are provided to explore the mechanisms of reactive tissue proliferation induced by abnormal movement of ischemic necrotic cores and pseudoarthrosis. However, the current level of evidence in this study is limited to exploratory observations. Although the results suggest that this multimodal strategy has potential reference value in specific clinical scenarios lacking MRI, current evidence is insufficient to confirm that it has become a mature and feasible alternative to MRI. In the future, large-scale, prospective clinical cohort studies will still be needed.

## Data Availability

The raw data supporting the conclusions of this article will be made available by the authors, without undue reservation.
